# Influence of Cyclic Heat Treatment Temperature on Microstructure and Mechanical Properties of 18Ni(C250) Maraging Steel

**DOI:** 10.3390/ma17122796

**Published:** 2024-06-07

**Authors:** Kai Xiao, Shun Han, Zhixin Li, Ruming Geng, Gaoyang Han, Yong Li, Chunxu Wang

**Affiliations:** 1 Shanghai Aircraft Design and Research Institute, Commercial Aircraft Corporation of China, Ltd., Shanghai 200232, China; xiaokai@comac.cc (K.X.); hangaoyang@comac.cc (G.H.); 2 Research Institute of Special Steels, Central Iron and Steel Research Institute, Beijing 100081, China; gengruming@nercast.com (R.G.); liyong@nercast.com (Y.L.); wangchunxu@nercast.com (C.W.); 3 School of Science, Civil Aviation Flight University of China, Guanghan 618307, China

**Keywords:** 18Ni(C250) maraging steel, cyclic heat treatment, microstructure evolution, mechanical properties

## Abstract

Cyclic heat treatment is an effective approach for enhancing the mechanical properties of 18Ni(C250) maraging steel, and the selection of cyclic heat treatment temperature is a key factor. In this study, a cyclic heat treatment process with a two-step solution treatment is employed to investigate the influence of cyclic heat treatment temperature, specifically the first solution treatment temperature (920 °C, 950 °C, and 980 °C), on the microstructure and mechanical properties of 18Ni(C250) maraging steel. The results indicate that with an increase in the cyclic heat treatment temperature, the average grain size of the 18Ni(C250) maraging steel decreases initially and then increases. When the cyclic heat treatment temperature reaches 950 °C, the grain size is at its minimum, exhibiting optimal grain uniformity. Additionally, the increase in cyclic heat treatment temperature results in a reduction in the size of martensitic lath with the same orientation inside the grains, along with an increase in the relative quantity of low-angle grain boundaries. Furthermore, the volume fraction and size of retained austenite show a monotonous increase with the rise in the temperature of the cyclic heat treatment, and the rate of increase becomes notably larger when the temperature is raised from 950 °C to 980 °C. Based on the observed microstructural changes, the variation in the mechanical properties of the 18Ni(C250) maraging steel was analyzed. Specifically, as the cyclic heat treatment temperature increases, the tensile strength of the 18Ni(C250) maraging steel initially increases and then stabilizes, while the elongation and fracture toughness exhibit a monotonic increase.

## 1. Introduction

Maraging steel is extensively applied in the fields of aviation, aerospace, and nuclear energy due to its excellent machinability and outstanding mechanical properties [1,2,3]. Its primary alloying element is nickel, typically present at levels above 14%. The elevated nickel content serves to lower the start temperature of the martensitic transformation, thereby facilitating the formation of finer lath-like martensite structures [4,5]. Furthermore, during the aging process, nickel reacts with iron to form an iron-nickel martensitic matrix, and the formation of a significant amount of precipitates requires the involvement of nickel in the reaction [6]. In addition to nickel, the maraging steel also contains cobalt, molybdenum, titanium, and other elements. Cobalt primarily solidifies within the martensitic matrix while promoting the precipitation of molybdenum in the form of nanoscale intermetallic compounds [7]. In maraging steel, the 18Ni(C250) maraging steel with 18% nickel content and a yield strength of 1700 MPa (250 ksi) is commonly used in the manufacturing of rocket engine casings and low-pressure turbine shafts for aircraft engines [8,9].

18Ni(C250) maraging steel is typically subjected to a heat treatment process involving solution treatment followed by aging in order to improve its strength and toughness. The purpose of the solution treatment is to dissolve low-melting-point carbides in the steel and form supersaturated metastable martensite during the quenching process, preparing for subsequent aging precipitation strengthening and eliminating rolling stresses [10]. The soaking temperature and cooling rate during the solution treatment are important factors that affect the mechanical properties of maraging steel. In a study by Li et al. [11], the influence of solution temperature ranging from 780 °C to 900 °C on the second-phase precipitation behavior and mechanical properties of T250 maraging steel ingots was compared. It was found that the highest density of nano-scale precipitates and the lowest lattice mismatch were achieved at a solution temperature of 820 °C, which contributed to increased elastic deformation and uniform elongation after yielding. Hou et al. [12] investigated the effect of solution temperature ranging from 750 to 1000 °C on the mechanical properties, grain size, and retained austenite content for Fe-9Cr-9Ni-5Co-2.5Mo maraging stainless steel. They observed that with increasing solution temperature, the original austenite grain size rapidly increased while the retained austenite content decreased. The presence of retained austenite can enhance the material’s low-temperature impact toughness. Regarding the influence of solution cooling rate, Tsuzaki et al. [13] investigated the impact of solution cooling rate on the martensitic lath morphology of a Fe-Ni maraging steel. Their results suggested that the initial martensitic lath appeared around the austenite grain boundaries, and the sizes of martensitic lath and blocks decreased with increasing cooling rate. The high-hardness austenite in this steel is formed through low-temperature solution treatment, which increases the resistance to the γ→α’ phase transformation during cooling and retains a significant amount of austenite, ensuring good low-temperature toughness of the tested steel. Morito et al. [14] compared the sizes of martensitic lath structures at different cooling rates in Fe-23Ni alloy and found that martensite with a single variant first formed at the original austenite grain boundaries and induced another variant in adjacent regions. Additionally, the sizes of martensitic lath blocks in furnace-cooled samples were larger than those in samples quenched in ice–salt water, but the sizes of corresponding sub-blocks remained unchanged. These studies indicate that solution heating temperature and cooling method are the main factors influencing the microstructure and mechanical properties of maraging steel after aging.

Compared with the single-step solution treatment process, the cyclic heat treatment process with multiple solution treatments can further refine the microstructure of 18Ni(C250) steel and enhance its mechanical properties [15]. The cyclic heat treatment process is based on the shear-induced martensitic phase transformation characteristic, which allows for thorough refinement of the original austenite grains in the material and facilitates complete recrystallization. The repeated cyclic phase transformations retain a significant number of microdefects within the martensite by means of reverse transformations, resulting in a noteworthy increase in the material’s stored energy. This elevated stored energy facilitates the fragmentation and refinement of laths, while also promoting recrystallization and grain refinement [16]. Moreover, Xie et al. [17] observed that after more than four cycles of phase transformation at the same temperature in T250 maraging steel, an abnormal enlargement of grain size occurred, and the strengthening effect of the cyclic phase transformation was not prominent. In their study of 18Ni(C250) maraging steel, Luo et al. [15] conducted a study on the influence of cyclic heat treatment with four cycles of solution treatment on grain size. Their findings revealed that implementing a cyclic heat treatment with progressively decreasing solution treatment temperatures proved to be an effective approach for refining the grain size of this steel. Additionally, the initial deformation plays a crucial role in determining the microstructure and properties of 18Ni(C250) maraging steel after cyclic phase transformation. Han et al. [18] found that effective grain refinement of 18Ni(C250) maraging steel grains and martensitic lath blocks can be achieved through a combination of forging deformation and cyclic phase transformation. With an increase in forging ratio, a grain-size number of 12 was attained, while the average size of martensitic lath blocks was refined to 7.09 μm.

These studies collectively demonstrate that cyclic heat treatment is an effective heat treatment process for refining the martensite microstructure. The number of cycles and the temperature combinations employed during each cycle are the primary factors influencing the final microstructure of maraging steel. Furthermore, in the cyclic heat treatment process, variations in the heating temperature during the initial solution treatment can result in changes in the density and distribution of microdefects within the transformed martensite, ultimately affecting the microstructure and mechanical properties of the maraging steel after subsequent solution treatment. Therefore, it is imperative to investigate the influence of cyclic heat treatment temperature on the microstructure and properties of C250 steel.

To this end, this study employed a cyclic heat treatment process consisting of two stages of solution treatment with different initial solution temperatures to investigate the influence of cyclic heat treatment temperature on the mechanical properties and microstructure of 18Ni(C250) maraging steel. In conjunction with this, the evolution of the microstructure, including the morphology and orientation of grain and martensitic lath, as well as the changes in mechanical properties, such as the tensile strength and fracture toughness of 18Ni(C250) maraging steel under different cyclic heat treatment temperatures, was investigated using metallographic observation, the Electron Backscatter Diffraction (EBSD) technique, and mechanical property testing.

## 2. Experimental Details

### 2.1. Materials and Heat Treatment Schedules

The experimental 18Ni(C250) maraging steel ingots were melted using a Vacuum Induction+Vacuum Arc Remelting process, and the composition is shown in Table 1. The steel ingots were subjected to hot extrusion deformation at 1080 °C and then air-cooled to room temperature after deformation. Subsequently, a solid solution treatment at 820 °C was performed to obtain the experimental steel rods.

**Figure 1:**
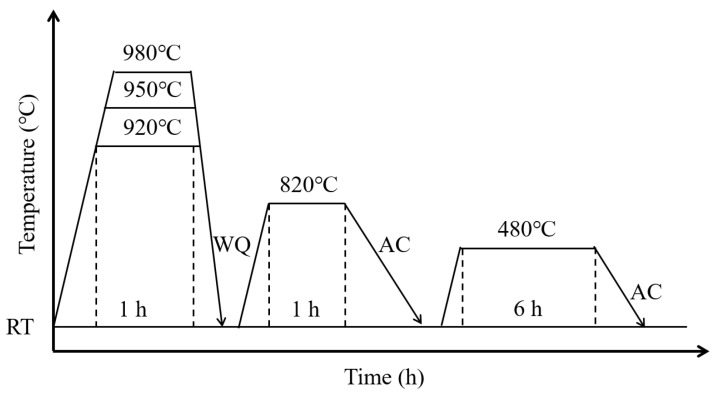
Cyclic heat treatment schedules for 18Ni(C250) maraging steel (RT: room temperature, WQ: water quenched, AC: air cooling).

The steel rods were cut into three sections and subjected to a cyclic heat treatment process that involved two cycles of solid solution treatment, as shown in Figure 1. The first solid solution was performed at heating temperatures of 920 °C, 950 °C, and 980 °C, followed by water quenching. The second solid solution was carried out at a lower temperature of 820 °C, followed by air cooling. Our previous research indicated that setting the final solution treatment temperature at 820 °C for cyclic heat treatment can lead to a more pronounced improvement in grain refinement. Finally, the cyclically heat-treated steels were subjected to an aging treatment at 480 °C for 6 h. In this way, four different heat treatment conditions were obtained for the 18Ni(C250) maraging steel, including one as-received state without cyclic heat treatment and three heat-treated states that underwent cyclic heat treatment at different temperatures followed by aging. The samples subjected to the first solid solution treatment at 920 °C, 950 °C, and 980 °C were denoted as 920Q-CHT, 950Q-CHT, and 980Q-CHT, respectively.

### 2.2. Material Tests

The samples for microstructural analysis were extracted from the core of 18Ni(C250) maraging steel rods after solid solution treatment at 820 °C, with the cross-sectional surface chosen as the observation area. The samples were polished and then electrolytic etching in a 10% chromic acid solution to create metallographic samples. Subsequently, the prepared metallographic samples were observed and photographed using a Zeiss 40MAT inverted metallographic microscope manufactured by Zeiss in Oberkochen, Germany. The average grain size was measured using the intercept method as specified in ASTM E112-2013 [19] and analyzed using the SISC-IAS8.0 image analysis software.

The crystal orientation of the alloy was analyzed using the EBSD technique. Sample preparation involved rough grinding with sandpaper, mechanical polishing, and electrolytic polishing. The electrolytic polishing was performed using a Struers Lectropol-5 electrolytic etching instrument (Struers, Ballerup, Denmark). Microstructural observations of the samples were conducted using a JSM-7001F field emission scanning electron microscope (JEOL Ltd., Tokyo, Japan) equipped with an EBSD system manufactured by EDAX. The scanning step size was set at 0.125 μm. The acquired EBSD data were processed and analyzed using the HKL-Channel5 software (https://speciation.net/Database/Instruments/Oxford-Instruments/HKL-Channel-5-;i1153) developed by Oxford Instruments (Abingdon, UK). The analysis focused on the characterization and distribution of grain boundaries and crystal orientations.

Samples were longitudinally extracted from the core of the steel rod for mechanical property testing at room temperature, wherein the length direction of the samples coincided with that of the steel rod. Tensile and impact experiments were conducted following the ASTM A307-21 standard [20]. The tensile samples have a diameter of 5 mm and a length of 25 mm. The impact samples measure 10 mm (width) × 10 mm (height) × 55 mm (length) and feature a V-notch. KIc determination is performed using standard three-point bending samples with dimensions of 5 mm (width) × 10 mm (height) × 55 mm (length), following the ASTM E399-2022 standard [21].

## 3. Results

### 3.1. Analysis of Grain Morphology and Size

**Figure 2:**
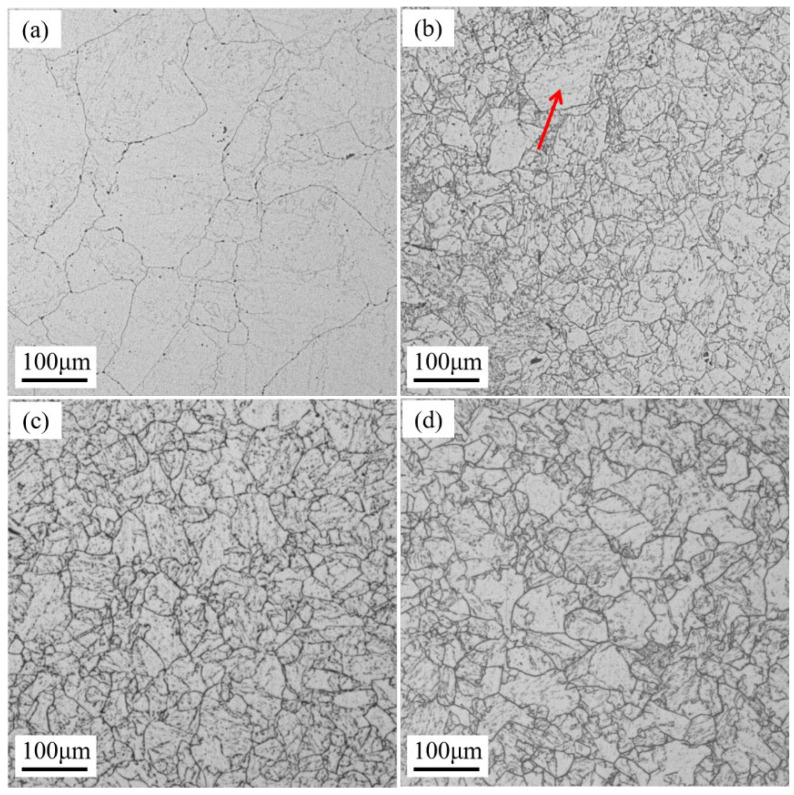
Metallographic diagrams of 18Ni(C250) steel under different heat treatment conditions: (**a**) as-received state; (**b**) 920Q-CHT, with a red arrow indicating coarse grain; (**c**) 950Q-CHT; and (**d**) 980Q-CHT.

Figure 2 shows the grain morphology of the 18Ni(C250) maraging steel in the as-received state and after cyclic heat treatment at different temperatures. As depicted in Figure 2a, the as-received 18Ni(C250) steel consists of a combination of coarse and fine grains, with larger grains measuring approximately 200 μm, corresponding to a grain-size number of 3.5 (Table 2). The presence of coarse grains can be attributed to the high hot forging temperature and low forging ratio at the core of the rod. Upon subjecting the as-received 18Ni(C250) steel to cyclic heat treatment, there is a noticeable decrease in grain size (Figure 2b–d), indicating that cyclic heat treatment effectively refines the coarse-grained structure at the core of the 18Ni(C250) steel after hot forging.

For cyclic heat treatment at different temperatures, the grain morphology and size also differ. At a cyclic heat treatment temperature of 920 °C (Figure 2b), an obvious mixed crystal phenomenon was observed in metallography, and the larger grain size was about 100 μm (the coarse grain indicated by the red arrow in Figure 2b), corresponding to a grain-size number of 7.5. This is mainly due to the mixed structure of fine and coarse grains in the initial grains of the as-received 18Ni(C250) steel, which leads to uneven nucleation during cyclic heat treatment and therefore produces mixed crystals. As the cyclic heat treatment temperature increases to 950 °C, the grain size further decreases and is more evenly distributed, as shown in Figure 2c. The resulting grain-size number is 8, which is equivalent to the grain-size number obtained from four rounds of cyclic heat treatment reported in the literature [15]. When the quenching temperature continues to increase to 980 °C, the average grain size becomes larger, and the grain-size number is 7.5. This is because the higher heating temperature causes the grain size to grow excessively and has a genetic influence on the size of austenite grains after the second solution treatment. In summary, cyclic heat treatment with decreasing temperature can effectively refine the grain size of 18Ni(C250) maraging steel after hot forging, and with increasing cyclic heat treatment temperature, the average grain size first decreases and then increases. At the quenching temperature of 950 °C, the grain size is smaller. In addition, the increase in cyclic heat treatment temperature is beneficial to improving the uniformity of grain size.

### 3.2. Evolution of Microstructure

**Figure 3:**
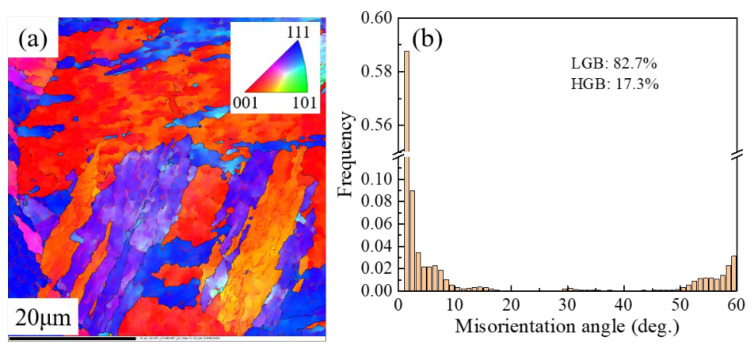

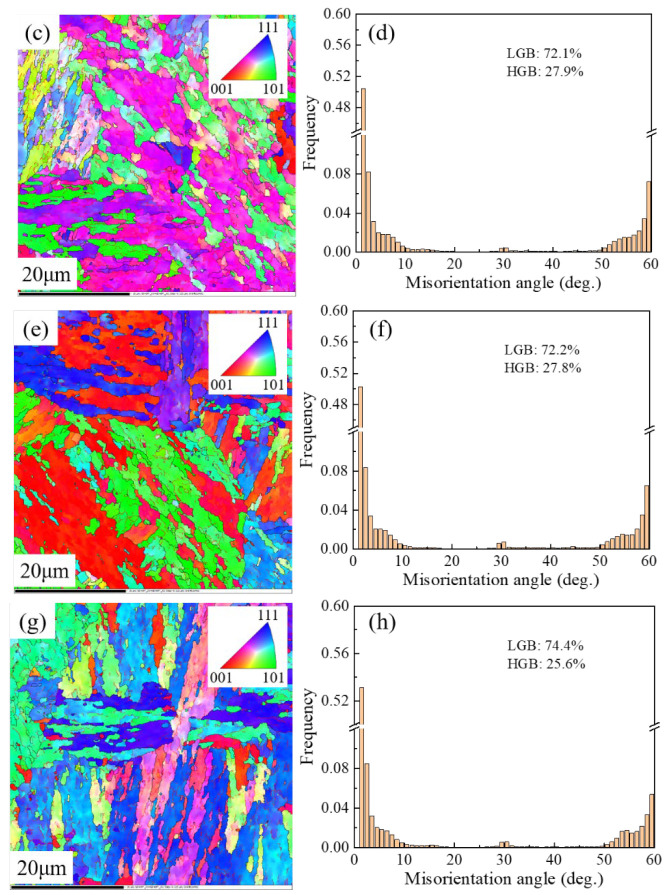
Orientation distribution map and histogram of grain boundary misorientation obtained based on EBSD for different samples of 18Ni(C250) maraging steel: (**a**,**b**) as-received state; (**c**,**d**) 920Q-CHT; (**e**,**f**) 950Q-CHT; (**g**,**h**) 980Q-CHT.

Figure 3 represents the EBSD orientation distribution maps and grain boundary misorientation histograms of 18Ni(C250) maraging steel in the as-received state and after cyclic heat treatment at different temperatures. The different colored regions in the figure represent the martensitic structure with different crystal orientations, while the black and red lines represent high-angle grain boundaries (>15°) and low-angle grain boundaries (<15°), respectively. From the orientation distribution map in Figure 3a and the inverse pole figure in the upper right corner of the figure, it can be observed that the microstructure of the as-received 18Ni(C250) maraging steel is coarse, with elongated martensitic laths (shown as blocks of the same color in Figure 3a). Furthermore, Figure 3a reveals the presence of a significant amount of low-angle grain boundaries within the laths, accounting for 82.7% of the total boundaries. The low-angle boundaries are possibly the block boundaries and subgrain boundaries [22].

After undergoing a cyclic heat treatment at 920 °C, the size of martensitic laths with the same orientation inside the grains significantly decreased. Furthermore, the percentage of low-angle grain boundaries (<15°) decreased from 82.7% (Figure 3b) to 72.1% (Figure 3d). Additionally, a large number of fine subgrains surrounded by low-angle grain boundaries were observed within the martensitic laths after being treated with this cyclic heat treatment temperature. As the cyclic heat treatment temperature increased, it can be seen from Figure 3d,f,h that the relative quantity of low-angle grain boundaries increased, indicating an increase in the number of subgrain boundaries per unit volume and a decrease in subgrain size. Moreover, from Figure 3c,e,g, it can be observed that as the temperature of the cyclic heat treatment increased, martensitic laths with the same orientation became more dispersed and their size decreased.

**Figure 4:**
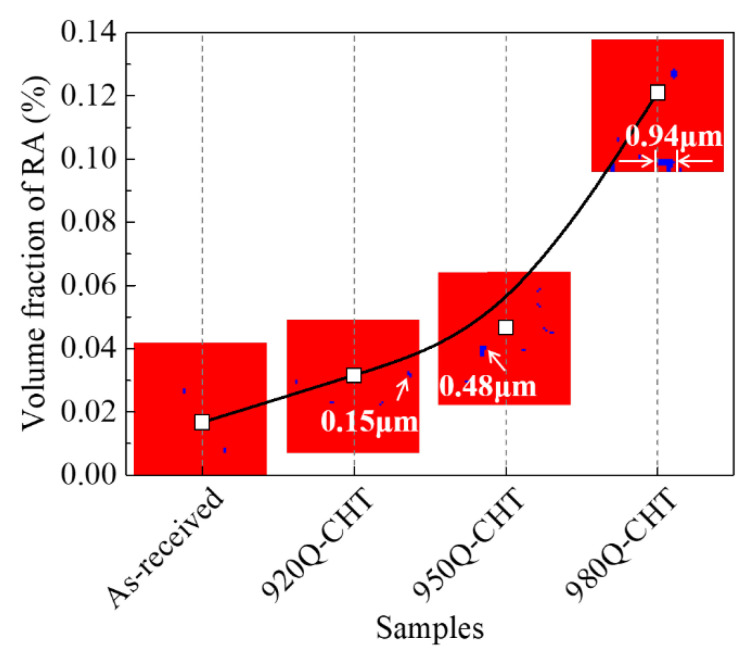
Variation in retained austenite volume fraction and size obtained based on EBSD in 18Ni(C250) maraging steel with cyclic heat treatment temperature.

Figure 4 illustrates the variation in retained austenite (RA) volume fraction and size in 18Ni(C250) maraging steel with different cyclic heat treatment temperatures. The white dots and curves represent the volume fraction, while the blue patches represent the morphology of retained austenite. From the figure, it can be observed that the volume fraction of retained austenite shows a monotonically increasing trend with the increase in cyclic heat treatment temperature. Moreover, the rate of increase becomes significantly higher when the temperature is raised from 950 °C to 980 °C. Additionally, as the water quenching temperature increases, the maximum size of retained austenite observed in the 18Ni(C250) maraging steel also increases, ranging from 0.15 μm to 0.94 μm. These results indicate that higher cyclic heat treatment temperatures lead to an increase in both the content and size of retained austenite in the final microstructure of 18Ni(C250) maraging steel.

### 3.3. Analysis of Mechanical Properties

**Figure 5:**
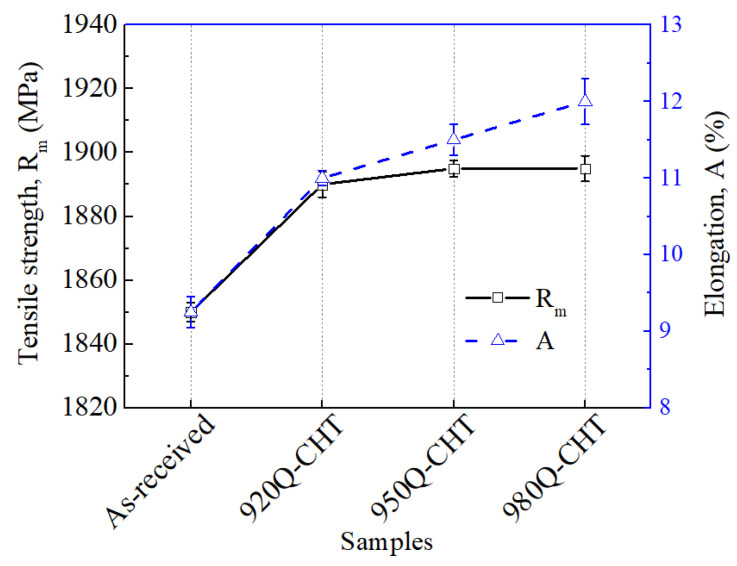
Variation in tensile strength and elongation of 18Ni(C250) maraging steel with changes in cyclic heat treatment temperature.

Figure 5 shows the tensile strength and elongation of 18Ni(C250) maraging steel in the as-received state and after cyclic heat treatment at different temperatures. It can be observed that both the strength and elongation increase significantly for the 18Ni(C250) maraging steel after cyclic heat treatment, indicating that cyclic heat treatment is beneficial for improving the mechanical properties of 18Ni(C250) maraging steel. As the cyclic heat treatment temperature increases from 920 °C to 950 °C, there is a slight increase in tensile strength. This is primarily due to further refinement in grain size, and an enhanced strengthening effect during subsequent aging processes because of the increase in solubility of alloy elements [11]. When the temperature continues to rise to 980 °C, although the solubility of alloy elements further increases, the strength does not continue to increase but tends to stabilize due to the slight increase in the average grain size.

The process of tensile fracture in metal materials involves the accumulation, growth, and propagation of damaged voids [23,24]. These voids tend to form and aggregate around insoluble second-phase inclusions, and their size determines the timing of void formation. Increasing the quenching temperature facilitates the dissolution of insoluble phases, delaying the occurrence of cracking. Additionally, as the quenching temperature increases, the martensite laths with the same orientation in 18Ni(C250) maraging steel become more dispersed and the lath size decreases (Figure 3c,e,g), making it more difficult for voids to grow and propagate, thereby further delaying fracture occurrence. Therefore, as shown by the blue dashed line in Figure 5, within the investigated range of temperatures, the elongation of 18Ni(C250) maraging steel tends to increase with higher cyclic heat treatment temperatures.

**Figure 6:**
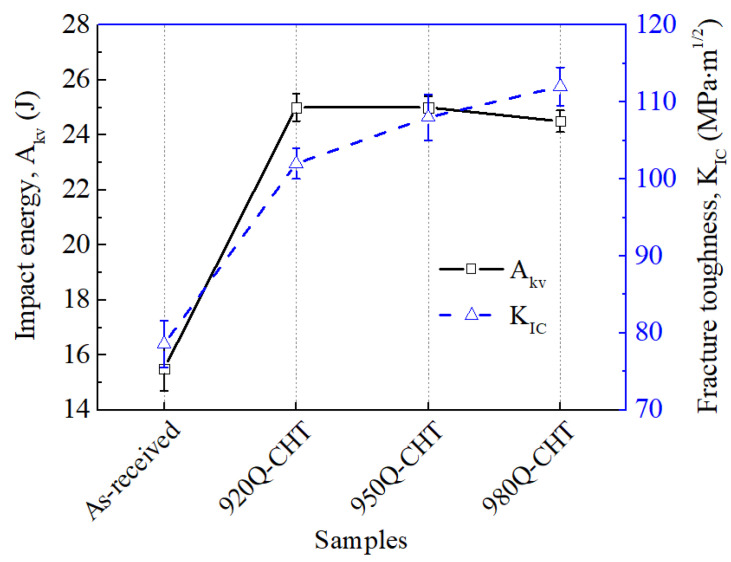
Variation in impact energy and fracture toughness of 18Ni(C250) maraging steel with cyclic heat treatment temperature.

Figure 6 shows the impact toughness and fracture toughness of 18Ni(C250) maraging steel. Impact toughness is a measure of the ability of high-strength steels to absorb plastic deformation energy and fracture energy under impact loading [25,26]. The black solid line in Figure 6 indicates that the impact toughness of 18Ni(C250) maraging steel significantly improves after cyclic heat treatment. Moreover, the change in impact toughness is not significant as the cyclic heat treatment temperature increases from 920 °C to 950 °C. This is because, after cyclic heat treatment at these two temperatures, the high-angle grain boundaries only change from 27.9% (Figure 3d) to 27.8% (Figure 3d). Since the high-angle grain boundary content is the primary factor affecting impact toughness, the energy absorbed by the steel during impact fracture does not change significantly. However, when the temperature rises to 980 °C, the high-angle grain boundaries decrease to 25.6%, and the grain size increases. As a result, the frequency of the large-angle turning of the crack propagation path decreases, reducing the energy consumed during crack propagation and leading to a slight decrease in impact energy.

Fracture toughness is an important physical property commonly used to assess the integrity and reliability of maraging steel structures, reflecting the combined effects of applied loads in the presence of defects [27]. The blue dashed line in Figure 6 indicates that the fracture toughness of 18Ni(C250) maraging steel increases rapidly after cyclic heat treatment, primarily due to the reduction in grain size [28,29]. It can be known from Figure 6 that, as the cyclic heat treatment temperature increases from 920 °C to 950 °C and 980 °C, the fracture toughness shows a continuously increasing trend.

## 4. Discussion

Cyclic heat treatment exploits the phenomenon of phase transformation recrystallization during the reverse transformation of martensite to austenite upon heating. By subjecting 18Ni(C250) maraging steel to cyclic phase transformations, the microstructure, including grains and martensitic laths, can be refined, consequently influencing the plastic deformation and fracture behavior. 18Ni(C250) maraging steel undergoes a martensitic transformation during water quenching, which is a rapid shear-type transformation. When this transformation occurs, the volume of different regions in the steel will undergo nonuniform expansion, producing obvious transformation internal stress, thereby increasing the number of microdefects such as dislocations inside the steel. When quenched at higher temperatures, these internal stresses and microdefects become more significant, providing greater nucleation energy for the austenite transformation during the second heating process and increasing the nucleation rate [16]. Therefore, as the cyclic heat treatment temperature increases from 920 °C to 950 °C, the grain size is reduced, and the coarse grains undergo further refinement, leading to a reduced degree of mixed crystals and a more uniform grain size distribution, as shown in Figure 2b,c. The reduction in grain size (Figure 2) is favorable for enhancing the grain boundary strengthening effect of 18Ni(C250) maraging steel [30], thus increasing the tensile strength of the steel (Figure 5). Moreover, when the grain size is reduced, the plastic deformation that occurs within the matrix under external forces can be accommodated by a larger number of grains. This leads to a more harmonized and uniform distribution of stress throughout the steel, thereby effectively enhancing its mechanical properties (Figure 5 and Figure 6). However, as the cyclic heat treatment temperature further increases to 980 °C, the grain size grows excessively, ultimately resulting in an increase in the size of grains after the second solution treatment. Therefore, the strength does not continue to increase (Figure 5), and the impact toughness decreases (Figure 6).

The increase in cyclic heat treatment temperature not only leads to a reduction in grain size but also results in a decrease in the size of martensitic laths (Figure 3). This is because the increased heating temperature results in a larger driving force for the transformation of martensitic laths during the quenching process, due to the increased degree of undercooling. Therefore, higher cyclic heat treatment temperatures can reduce the size of subgrains and refine the martensitic microstructure, both of which have a significant impact on the fracture toughness of the material [31]. Moreover, with increasing the cyclic heat treatment temperature, the distribution of martensite lathes with the same orientation becomes more dispersed, and there is a relatively greater proportion of low-angle grain boundaries (as shown in Figure 3). This increases the resistance to crack propagation within the grains, requiring more energy for the crack to extend the same distance [32]. Thus, as the cyclic heat treatment temperature increases from 920 °C to 950 °C and 980 °C, both the elongation and fracture toughness show continuously increasing trends (Figure 5 and Figure 6).

Additionally, when the first solid solution treatment temperature is higher, the austenite in the steel is less prone to undergo martensitic transformation during the water quenching process. As a result, a greater volume fraction and larger size of retained austenite are preserved. These retained austenite phases remain relatively stable during the subsequent solution treatment process, which further increases the volume fraction and size of retained austenite after the second treatment (as shown in Figure 4). The increases in retained austenite are considered beneficial for improving the fracture toughness [33,34], reducing the degree of stress concentration at the crack tip and thereby slowing down crack propagation. Thus, it can be seen from Figure 6 that the fracture toughness is significantly improved with the increase in cyclic heat treatment temperature.

## 5. Conclusions

The effects of cyclic heat treatment temperature (920–980 °C) on the microstructure and mechanical properties of 18Ni(C250) maraging steel were studied through experiments. Based on the obtained results, the following conclusions can be drawn:

(1) The cyclic heat treatment recommended in this study effectively refines the microstructure of 18Ni(C250) maraging steel after hot forging and improves its mechanical properties. With an increase in the cyclic heat treatment temperature, the average grain size of 18Ni(C250) maraging steel decreases initially and then increases. When cyclic heat treatment at the temperature of 950 °C, the grain size is the smallest, indicating the best grain uniformity.

(2) With an increase in the cyclic heat treatment temperature, the martensite laths with the same orientation inside the grains become more dispersed and smaller in size. Additionally, there is an increase in the relative quantity of low-angle grain boundaries and a reduction in subgrain size. Moreover, the volume fraction and size of retained austenite exhibit a monotonic increasing trend with an increase in the cyclic heat treatment temperature, and when the temperature is raised from 950 °C to 980 °C, the rate of increase significantly amplifies.

(3) With an increase in the cyclic heat treatment temperature, the tensile strength of 18Ni(C250) maraging steel initially increases and then stabilizes, while the elongation monotonically increases. When the cyclic heat treatment temperature is raised from 920 °C to 950 °C, the impact toughness of the steel shows no significant change. However, when the temperature is further increased to 980 °C, the impact toughness of 18Ni(C250) maraging steel slightly decreases due to the reduction in high-angle grain boundaries. As the cyclic heat treatment temperature increases, the fracture toughness exhibits a continuously increasing trend. This is mainly attributed to the increased resistance to crack propagation resulting from the more dispersed distribution of the martensite lathes, as well as the reduced stress concentration at the crack tip due to the higher volume fraction and larger size of retained austenite.

## Figures and Tables

**Table 1:** Main element composition of 18Ni(C250) maraging steel.

Element	C	Si	Mn	P	S	Cr	Ni	Cu	Mo
wt%	0.0045	<0.05	<0.01	0.0022	0.0004	0.019	18.01	<0.01	4.92
Element	Ti	AI	As	Bi	Co	Fe			
wt%	0.40	0.12	0.0022	0.00003	7.72	Remainder			

**Table 2:** Grain size and grain-size number measured by intercept method under different heat treatment conditions.

Heat Treatment States	Grain Size (μm)	Grain-Size Number
As-received state	91.8	3.5 ± 0.5
920 °C × 1 hWQ + 820 °C × 1 hAC	24.5	7.5 ± 0.5
950 °C × 1 hWQ + 820 °C × 1 hAC	22.3	8 ± 0.5
980 °C × 1 hWQ + 820 °C × 1 hAC	25.0	7.5 ± 0.5

## Data Availability

Data are contained within the article.
